# Cofilin-1 is an essential redox sensor for NLRP3 inflammasome activation

**DOI:** 10.1186/1546-0096-13-S1-O52

**Published:** 2015-09-28

**Authors:** YH Park, D Kastner, JJ Chae

**Affiliations:** 1 NIH/NHGRI, Bethesda, MD, USA

## Introduction

NLRP3 has a pivotal role in nucleating the inflammasome, a cytoplasmic multiprotein complex that mediates the maturation of the proinflammatory cytokine interleukin-1β (IL-1β) by activating caspase-1. Mutations in the gene encoding NLRP3 cause a spectrum of autoinflammatory disease, the cryopyrin-associated periodic syndromes (CAPS). It has been reported that generation of reactive oxygen species (ROS) is a major NLRP3 inflammasome-activating factor. However, the molecular mechanism by which a change in cellular redox state leads to NLRP3 inflammasome activation has not been elucidated. Here we show that cofilin-1, a redox sensitive actin binding protein, is involved in NLRP3 inflammasome activation.

## Objectives

To investigate how ROS activates the NLRP3 inflammasome.

## Methods

Cell culture supernatants from bone marrow derived macrophages (BMDMs) of wild-type or NLRP3-KO mice were analyzed by mass spectrometry. Inflammasome activation was analyzed by Western blotting of secreted IL-1β or ASC oligomerization. The interaction of NLRP3 with cofilin-1 was examined by co-immunoprecipitation from BMDMs or transfected cells.

## Results

Cofilin-1 is highly secreted along with IL-1β from LPS-primed BMDMs in response to the known NLRP3 activator, ATP, whereas knockdown of cofilin-1 reduces NLRP3 inflammasome activation. Cofilin-1 directly interacts to the nucleotide-binding domain (NBD) of the NLRP3 protein in LPS-primed BMDMs. However, cofilin-1 is dissociated from NLRP3 in a ROS-sensitive manner when the cells are stimulated with the NLRP3 inflammasome activators, ATP or nigericin, which induce oxidation of cofilin-1. Indeed, the interaction of cofilin-1 with NLRP3 is increased significantly when the oxidation site of cofilin-1 is substituted from cysteine to alanine. Moreover, the assembly of inflammasome components is impaired in cells expressing oxidation-resistant mutant cofilin-1.

## Conclusion

Taken together, these findings suggest that cofilin-1 is a key component in regulating the NLRP3 inflammasome in response to ROS. In addition, our data suggest a potential target for the inflammatory conditions involving the NLRP3 inflammasome, including gout, type 2 diabetes mellitus, atherosclerosis, and Alzheimer's disease.

